# Clinical utility of circulating tumor cell PD-L1 expression for predicting immunotherapeutic response and prognosis in esophageal cancer

**DOI:** 10.1016/j.jlb.2026.100681

**Published:** 2026-10-21

**Authors:** Jing Li, Yueze Li, Lin Chen, Zhonglin Yang, Yue Lu, Dongjiang Tang, Yanru Qin

**Affiliations:** a Department of Oncology, The First Affiliated Hospital of Zhengzhou University, longhu zhonghuan road, No.1, Zhengzhou, Henan, 450000, P. R. China; b Zhuhai Sanmed Biotech Ltd., 266 Tongchang Road, Zhuhai, Guangdong, 519060, P. R. China

**Keywords:** Esophageal cancer, Circulating tumor cells, PD-L1, Immunotherapy, Predictive biomarker, Liquid biopsy

## Abstract

**Background:**

Immune checkpoint inhibitors (ICIs) have improved outcomes in esophageal cancer, yet reliable biomarkers remain lacking. Tissue PD-L1 testing is limited by intratumoral heterogeneity and invasiveness, whereas circulating tumor cells (CTCs) offer a non-invasive alternative. This study evaluated CTC PD-L1 expression as a predictive and prognostic biomarker in patients with advanced esophageal cancer receiving chemo-immunotherapy.

**Methods:**

A total of 93 patients with stage III–IV esophageal cancer were enrolled. The primary biomarker analysis was restricted to 59 patients who received chemo-immunotherapy, while 34 patients treated with chemotherapy alone were analyzed separately. Baseline CTCs were isolated and PD-L1 expression assessed, alongside tissue PD-L1 (tPD-L1) by immunohistochemistry. Associations with objective response rate (ORR), progression-free survival (PFS), and overall survival (OS) were evaluated using ROC curves and univariate and multivariable Cox regression.

**Results:**

CTC positivity correlated significantly with advanced stage, metastasis (*p* = 0.0411), and larger tumor size (*p* = 0.0454). In the chemo-immunotherapy cohort, CTC PD-L1 positivity was associated with a higher ORR (75.0% vs. 29.6%, *p* < 0.001) and showed moderate predictive ability (AUC = 0.7049, *p* = 0.0037). CTC PD-L1 positivity was significantly associated with OS (HR = 0.254, *p* = 0.002), but not significantly with PFS in univariate analysis (HR = 0.546, *p* = 0.042; Kaplan–Meier *p* = 0.061). Conversely, tPD-L1 status was not significantly associated with PFS or OS (both *p* > 0.05). In multivariable analysis adjusting for age, sex, histological subtype, stage, metastasis, and treatment modality, CTC PD-L1 remained an independent prognostic factor for PFS (HR = 0.488, *p* = 0.039) and OS (HR = 0.244, *p* = 0.002).

**Conclusions:**

Baseline CTC PD-L1 expression was associated with favorable response and OS in patients receiving chemo-immunotherapy, and may complement tissue testing. These exploratory findings require prospective validation in larger cohorts before clinical use.

## 1 Introduction

Esophageal cancer ranks as the seventh most common malignancy and the sixth leading cause of cancer-related mortality worldwide, with esophageal squamous cell carcinoma (ESCC) representing the predominant histological subtype, particularly in East Asia [1]. Despite advances in multimodal treatment approaches, the prognosis for patients with advanced-stage disease remains poor, with limited therapeutic options and suboptimal long-term survival outcomes [2], [3].

The emergence of immune checkpoint inhibitors (ICIs) targeting the programmed death-1 (PD-1) and programmed death-ligand 1 (PD-L1) axis has revolutionized the therapeutic landscape for esophageal cancer. Landmark phase III trials, including KEYNOTE-181 [4], ESCORT [5], and KEYNOTE-590 [6], have demonstrated promising efficacy and manageable safety profiles for ICIs in advanced esophageal cancer, leading to their integration into clinical practice across multiple lines of therapy. However, the clinical benefit of immunotherapy remains highly heterogeneous, with only a subset of patients achieving durable responses, while others derive minimal benefit or experience immune-related adverse events [7], [8]. This variability underscores the urgent need for reliable predictive biomarkers to identify patients most likely to respond to ICI-based treatment and to guide personalized therapeutic decision-making.

Currently, tumor tissue PD-L1 expression, assessed by immunohistochemistry (IHC) using the combined positive score (CPS), remains the most widely utilized biomarker for predicting ICI efficacy in esophageal cancer [9]. However, its clinical utility is constrained by several fundamental limitations. First, PD-L1 expression exhibits substantial intratumoral spatial heterogeneity, with significant variation across different regions of the same tumor, rendering limited biopsy specimens potentially unrepresentative of the overall PD-L1 status [7], [10], [11]. Multi-region sampling studies have demonstrated that PD-L1 expression can vary considerably between tumor regions, with regional discordance rates exceeding 60% in esophageal cancer [10], [12]. Second, PD-L1 expression is dynamically regulated and can be modulated by therapeutic interventions, prior treatments, and evolving immune pressure, making it a temporally unstable biomarker. Third, tissue biopsies are invasive, carry procedural risks, and are often logistically challenging to obtain, particularly in advanced disease settings where serial monitoring is clinically desirable. These limitations have prompted the exploration of alternative, minimally invasive biomarkers capable of capturing real-time tumor biology.

Liquid biopsy approaches, particularly the detection and characterization of circulating tumor cells (CTCs), offer a promising non-invasive strategy that may complement tissue-based testing [13], [14], [15]. CTCs are tumor cells shed from primary and metastatic sites into the peripheral circulation, and have been proposed as a potentially more integrated assessment of tumor heterogeneity compared with a single tissue biopsy [16]; however, direct evidence that CTC analysis fully captures intratumoral heterogeneity is still limited. Importantly, PD-L1 expression can be evaluated on CTCs (CTC PD-L1), enabling dynamic monitoring of PD-L1 status through serial blood sampling [17]. Emerging evidence in other malignancies, including non-small cell lung cancer (NSCLC) and hepatocellular carcinoma, has demonstrated the predictive potential of CTC PD-L1 for immunotherapy outcomes, with several studies reporting significant associations between baseline CTC PD-L1 positivity and superior objective response rates (ORR) as well as prolonged progression-free survival (PFS) and overall survival (OS) [18], [19].

Despite these promising findings, several critical gaps remain in the current understanding of CTC PD-L1 in esophageal cancer. First, most existing studies on CTC PD-L1 have been conducted in NSCLC, with limited data available specifically for esophageal cancer, particularly in the context of chemo-immunotherapy. Second, prior investigations have largely focused on either the predictive value for short-term response or the prognostic value for long-term survival, but few have systematically integrated both endpoints within the same patient cohort to provide a comprehensive evaluation. Third, the comparative performance of CTC PD-L1 relative to conventional tissue PD-L1 testing and total CTC enumeration has not been systematically evaluated in esophageal cancer, leaving uncertainty as to which biomarker offers the greatest clinical utility. Fourth, most published studies have employed small sample sizes and lacked rigorous ROC curve analyses with identified optimal cutoffs, limiting the clinical translatability of their findings. Finally, the dynamic relationship between CTC PD-L1 expression and clinicopathological parameters—such as T stage, metastatic burden, and tissue PD-L1 status—remains inadequately characterized in esophageal cancer patients receiving immunotherapy-based regimens.

To address these knowledge gaps, the present study was designed to comprehensively evaluate the clinical utility of baseline CTC PD-L1 expression as a predictive and prognostic biomarker in patients with stage III–IV esophageal cancer receiving chemo-immunotherapy. Specifically, we sought to (1) investigate the associations of CTC and CTC PD-L1 status with clinicopathological parameters, including tumor size, clinical stage, and metastatic status; (2) compare the predictive value of CTC PD-L1, tPD-L1, and total CTC burden for objective response in patients receiving chemo-immunotherapy; and (3) assess the prognostic significance of these biomarkers for PFS and OS using Kaplan–Meier survival analyses and univariate and multivariable Cox regression. We hypothesized that CTC PD-L1 expression would demonstrate better predictive and prognostic performance than tissue-based tPD-L1 testing and total CTC enumeration, offering a more dynamic and representative approach to guide immunotherapy decisions in esophageal cancer.

## 2 Materials and methods

1. Study design

This study was a non-interventional, prospective, real-world investigation conducted at the First Affiliated Hospital of Zhengzhou University (Ethics approval number 2024-KY-0100-002), enrolling patients with advanced esophageal cancer without autoimmune diseases. Participants received at least two cycles of chemotherapy combined with PD-1/PD-L1 blockade immunotherapy starting from April 2024. Patients who received chemotherapy alone were not included in the primary biomarker analysis and were analyzed separately as an exploratory reference group. Peripheral blood samples (10 mL) were collected at baseline (T0) for CTC detection and PD-L1 expression analysis at Zhuhai Sanmedbio (Fig. 1), the effects of response (ORR, PFS, and OS) to combination of chemotherapy and immune checkpoint inhibitor (ICI) therapy and follow-up care are being studied at the First Affiliated Hospital of Zhengzhou University with more than 3 months.

2. Detection and characterization of circulating tumor cells and tissue PD-L1 expression

**Fig. 1:**
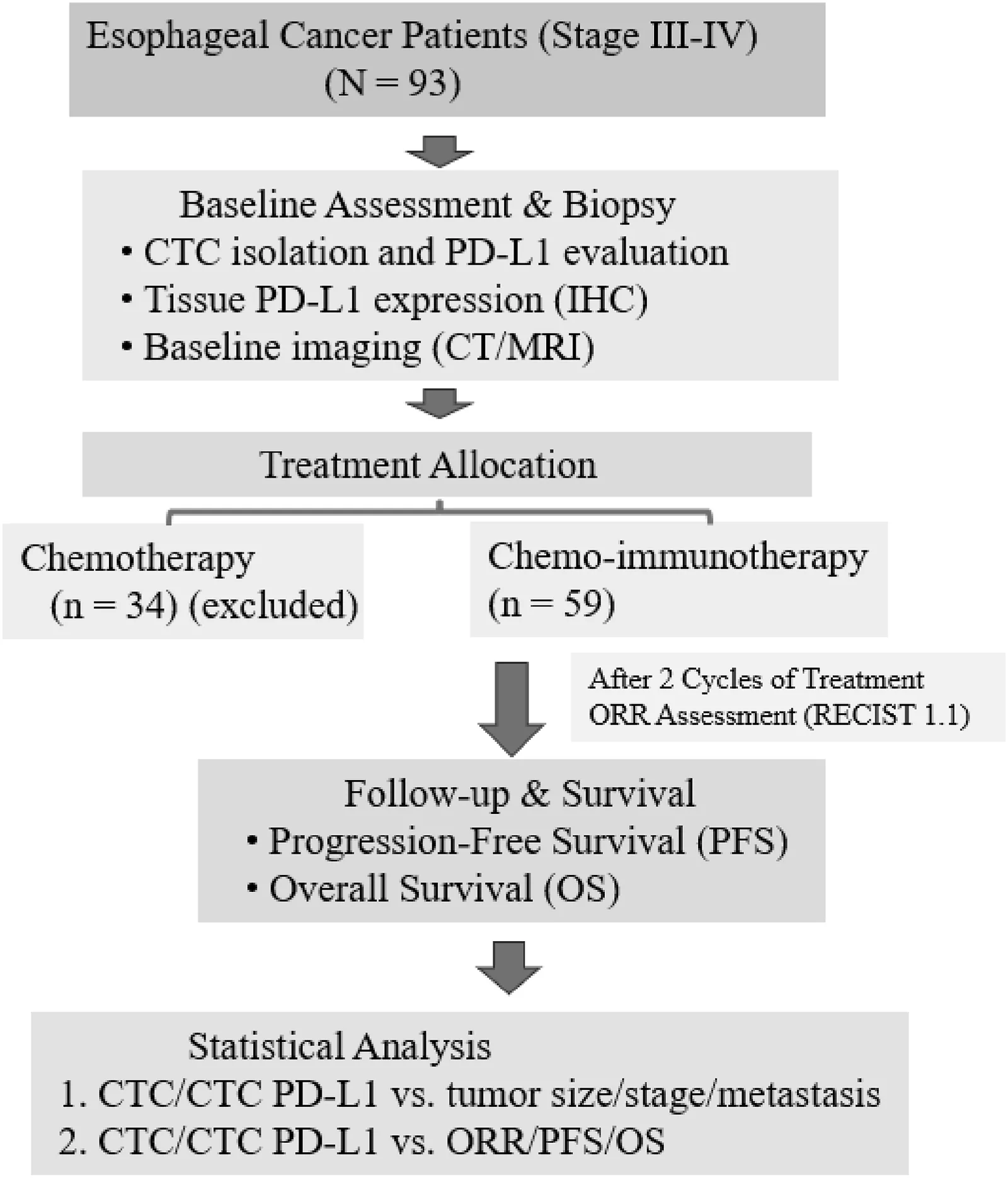
**Study workflow and patient disposition.** A total of 93 patients with stage III–IV esophageal cancer was enrolled in this study. Baseline assessments included CTC isolation with PD-L1 evaluation, tissue PD-L1 expression detection by immunohistochemistry (IHC), and baseline imaging (CT/MRI). Patients were subsequently allocated to receive either chemotherapy alone (n = 34) or chemo-immunotherapy (n = 59). Follow-up was conducted to assess PFS and OS. Statistical analyses were performed to evaluate (1) associations of CTC and CTC PD-L1 with tumor size, clinical stage, and metastasis status, and (2) correlations of CTC and CTC PD-L1 with ORR, PFS, and OS.

The LiquidBiopsy™ system (Zhuhai Sanmed Biotech Ltd., Zhuhai, China) was employed for CTC isolation and enrichment, as detailed in Winer J P et al. [20]. In brief, PBMCs were separated from 10 mL of whole blood by Ficoll (ρ = 1.077 g/mL; Solarbio, Cat: P8610) gradient centrifugation at 1000×g for 10 min at room temperature, fixed in 2% paraformaldehyde (Solarbio, Cat: P1110) for 1 h at room temperature, and subsequently incubated with capture cocktail reagents and streptavidin beads (BD Biosciences, Cat: 557812) to immobilized CTCs. The capture cocktail consisted of biotinylated anti-EpCAM (Santa Cruz Biotechnology, Cat: sc-59906; Clone: HEA125)/anti-HER2 (Santa Cruz Biotechnology, Cat: sc-23864; Clone: 24D2)/anti-Trop2 antibody (Hubio, Cat: HA600011; Clone: A5C5), each at a final concentration of 1 μg/mL. For phenotypic characterization, cells were stained with a panel detection kit (Zhuhai Sanmed Biotech Ltd.) consisting of anti-pan CK (Clone: C11), anti-PD-L1 (Clone:28-8), and anti-CD45 (Clone: 2D1) antibodies (each at a final concentration of 4 μg/mL) overnight at 4 °C, with DAPI (Thermo Fisher, Cat: D1306) used for nuclear visualization. To evaluate PD-L1 status, CTCs were incubated with HRP-labeled anti-PD-L1 antibody, followed by anti-HRP IgG Alexa Fluor® 594 secondary antibody (Jackson ImmunoResearch, Cat: 123-585-021). CTCs were identified based on specific phenotypes with threshold of CK mean of fluorescence intensity (MFI) and ratio of CK/CD45, while PD-L1 positivity was defined based on thresholds for both PD-L1 MFI and the PD-L1/CD45 ratio. A patient was categorized as CTC PD-L1 positive upon detection of at least one PD-L1-expressing CTC, whereas CTC positivity was assigned when at least one CK-positive CTC was identified [17]. Tissue PD-L1 expression was examined with 22C3 pharmDx (Dako) and defined as positive at threshold of TPS ≥ 10% [21].

3. Response assessment and survival endpoints

Radiologic tumor response was evaluated per RECIST 1.1 guidelines following two cycles of treatment. ORR was calculated as the proportion of patients achieving either complete response (CR) or partial response (PR) relative to the total patient population. PFS was defined as the interval from commencement of chemo-immunotherapy combination to the earliest occurrence of radiographic disease progression or mortality from any cause-with death, regardless of whether preceded by documented progression. OS was measured from the same treatment initiation point to the date of death following enrollment.

4. Statistical analysis of CTC biomarkers and survival outcomes

CTC enumeration and PD-L1 profiling were performed to characterize the patient cohort. Subjects were classified as CTC PD-L1^+^ (≥1 PD-L1-positive CTC detected) or CTC PD-L1^−^ (none detected). Receiver operating characteristic (ROC) curve analysis was used to evaluate the discriminative ability of each biomarker, and the optimal cutoff was determined by the Youden index. Univariate and multivariable Cox proportional hazards regression models were used to evaluate the prognostic value of CTC PD-L1 status for PFS and OS. Variables included in the multivariable model were selected a priori based on clinical relevance and included age, sex, histological subtype, clinical stage, metastasis status, and treatment modality; the number of events per variable was checked to avoid overfitting. Kaplan–Meier curves were compared with the Mantel–Cox test. Analyses were conducted with GraphPad Prism 8.2.0 and SPSS 25, with *p* < 0.05 (two-sided) denoting significance.

## 3 Results

1. **Clinicopathological Correlates of CTC and CTC PD-L1 Positivity**

A total of 93 patients were enrolled, of whom 63 (67.7%) were CTC-positive and 50 (53.8%) were CTC PD-L1-positive. Among them, 59 patients received first-line of chemo-immunotherapy and constituted the primary analysis cohort; 34 patients received chemotherapy alone and were analyzed separately. The distribution of CTC counts and CTC PD-L1 counts across the overall cohort and clinicopathological subgroups is summarized in Supplementary Table S1. In the overall cohort, the median CTC count was 3 (IQR 1–5; range 0–167) and the median CTC PD-L1 count was 1 (IQR 0–3; range 0–159). Both CTC and CTC PD-L1 counts were higher in patients with stage IV disease (median 4, IQR 1–7 and median 2, IQR 0–4, respectively) and in those with metastasis (median 4, IQR 1–7 and median 2, IQR 0–4) than in patients with stage III disease (median 1, IQR 0–3 and median 0, IQR 0–1) or without metastasis (median 1, IQR 0–3 and median 0, IQR 0–1), whereas no obvious differences were observed by sex or cancer type. The distribution of CTC and CTC PD-L1 positivity across clinicopathological subgroups is summarized in Table 1. Regarding clinical stage, the CTC-positive rate increased progressively from stage III (53.6%) to stage IV (78.8%). A similar upward trend was observed for CTC PD-L1 positivity (stage III: 39.0%; stage IV: 65.4%). In terms of metastasis, patients with M_1_ (≥1 metastatic site) exhibited significantly higher CTC counts than M_0_ patients (*p* = 0.0411, Fig. 2A), with a cutoff of 3 yielding a sensitivity of 73.47% and specificity of 68.18% (AUC = 0.7481, 95% CI: 0.6481–0.8482, *p* < 0.0001; Fig. 2E). Although CTC PD-L1 counts did not reach statistical significance between the two groups (*p* = 0.1591, Fig. 2B), the positivity rate was higher in M_1_ patients (67.3% vs. 38.6%), and a cutoff of 2 provided a sensitivity of 59.18% and specificity of 72.73% (AUC = 0.6818, 95% CI: 0.5731–0.7905, *p* = 0.0026; Fig. 2F).

**Table 1:** Clinicopathological characteristics and CTC positivity in patients (n = 93).

Characteristic	N	CTC^+^	CTC PD-L1^+^
Total	93	63 (67.7%)	50 (53.8%)
Gender			
Female	32	20 (62.5%)	16 (50.0%)
Male	61	43 (70.5%)	34 (55.7%)
Stage			
III	41	22 (53.6%)	16 (39.0%)
IV	52	41 (78.8%)	34 (65.4%)
Cancer type			
Adenocarcinoma	14	7 (50.0%)	6 (42.9%)
Squamous cell carcinoma	79	56 (70.9%)	44 (55.7%)
Tumor size			
T1-2	39	19 (48.7%)	14 (35.9%)
T3-4	54	44 (81.8%)	36 (66.7%)
Metastasis			
M0	44	24 (54.5%)	17 (38.6%)
M_X_	49	39 (79.6%)	33 (67.3%)
tPD-L1			
<10%	44	28 (63.6%)	21 (46.5%)
≥10%	49	35 (71.4%)	29 (59.2%)

**Fig. 2:**
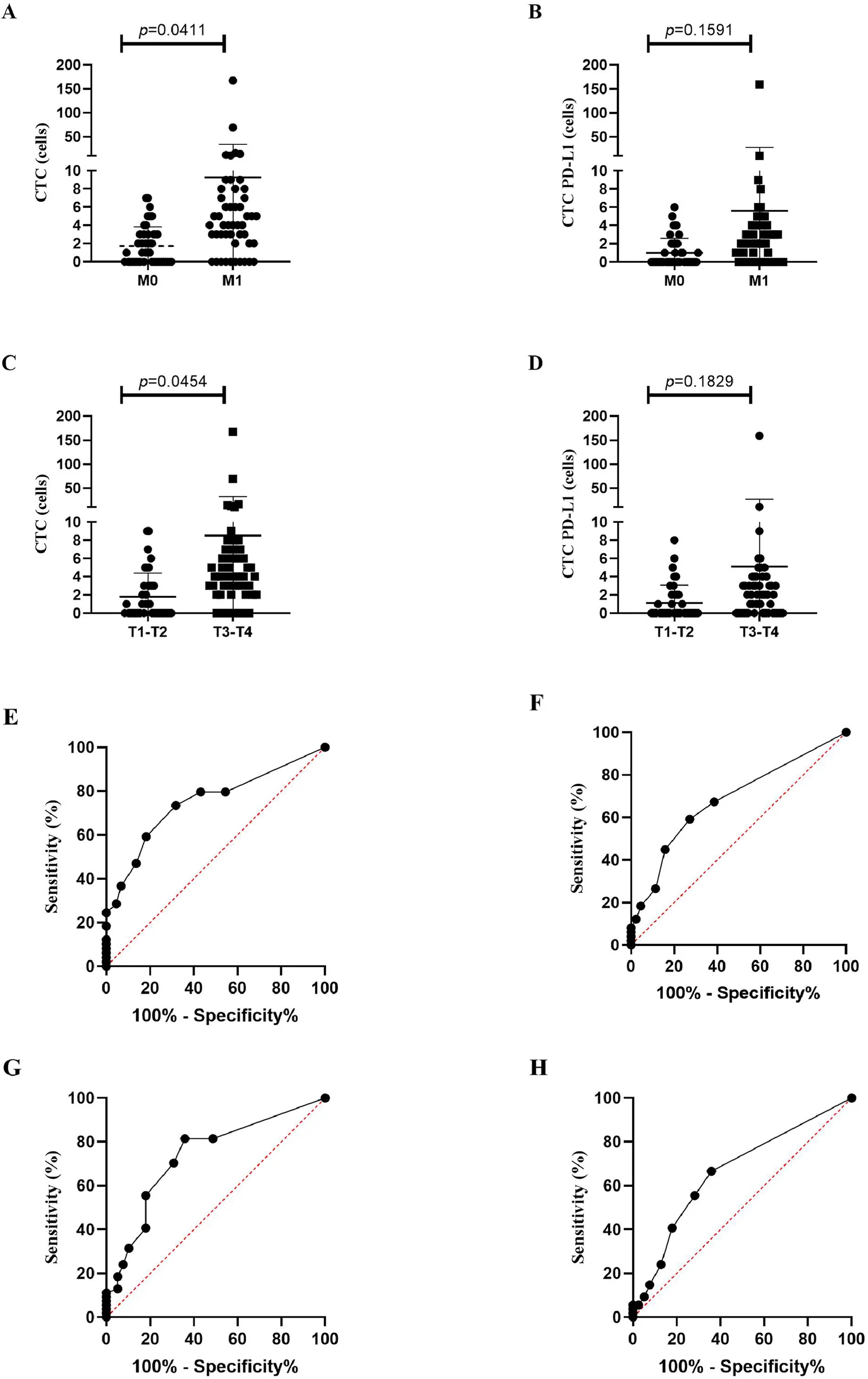
**Baseline CTC and CTC PD-L1 levels stratified by metastasis and T stage, with ROC curve analyses for diagnostic performance.** (A–D) Box-scatter plots comparing total CTC counts (A) and CTC PD-L1 counts (B) between M0 and Mx patients, as well as total CTC counts (C) and CTC PD-L1 counts (D) between T1–T2 and T3–T4 subgroups. Statistical comparisons were performed using the Mann–Whitney *U* test, with *p*-values of 0.0411, 0.1591, 0.0454, and 0.1829, respectively. (E–H) Receiver operating characteristic (ROC) curves evaluating the diagnostic performance of total CTC and CTC PD-L1 counts for distinguishing metastatic status (E, CTC; F, CTC PD-L1) and TNM stage (G, CTC; H, CTC PD-L1).

Tumor size was also associated with CTC detection. Patients with T_3–4_ tumors had markedly higher CTC positivity (81.8% vs. 48.7%) and significantly elevated CTC counts (*p* = 0.0454, Fig. 2C) compared with those with T_1–2_ tumors. At a cutoff of 3, the sensitivity and specificity for distinguishing T_3–4_ from T_1–2_ were 70.37% and 69.23%, respectively (AUC = 0.7360, 95% CI: 0.6316–0.8403, *p* = 0.0001; Fig. 2G). Similarly, CTC PD-L1 positivity was higher in the T_3–4_ group (66.7% vs. 35.9%), though the difference in CTC PD-L1 counts did not reach statistical significance (*p* = 0.1829, Fig. 2D); a cutoff of 1 yielded a sensitivity of 66.67% and specificity of 64.10% (AUC = 0.6636, 95% CI: 0.5511–0.7761, *p* = 0.0073; Fig. 2H). Finally, when stratified by tumor PD-L1 expression, though patients with tPD-L1 ≥10% showed slightly higher rates of both CTC positivity (71.4% vs. 63.6%) and CTC PD-L1 positivity (59.2% vs. 46.5%) compared with those with tPD-L1 <10%, spearman's correlation analysis showed no significant association between tPD-L1 and total CTC counts (rho = 0.091, *p* = 0.396), nor between tPD-L1 and CTC PD-L1 expression (rho = 0.136, *p* = 0.200) (Supplementary Table S2). These findings suggest that tissue PD-L1 status and CTC-derived PD-L1 assessment may provide independent or complementary information, rather than reflecting the same biological signal; however, this hypothesis requires more direct validation with paired tissue–CTC samples.

2. **Correlations between CTC PD-L1, tPD-L1, Total CTC Burden and Immunotherapy Clinical Response**

We stratified patients by CTC PD-L1 status and tPD-L1 expression to compare ORR in the chemo-immunotherapy cohort (n = 59). Patients with CTC PD-L1-positive status exhibited a markedly higher proportion of PR compared with CTC PD-L1-negative individuals (75.0% vs 29.6%; Fig. 3A), the latter of whom predominantly presented with SD/PD. In contrast, tissue-based tPD-L1 stratification did not reveal a clear divergence in ORR distribution between tPD-L1-positive and tPD-L1-negative subgroups (54.8% vs 53.5%; Fig. 3B). When categorized by baseline CTC positivity, CTC-negative patients showed a larger fraction of PR, whereas CTC-positive cases were more likely to experience SD/PD (73.3% vs 31.6%; Fig. 3C).

**Fig. 3:**
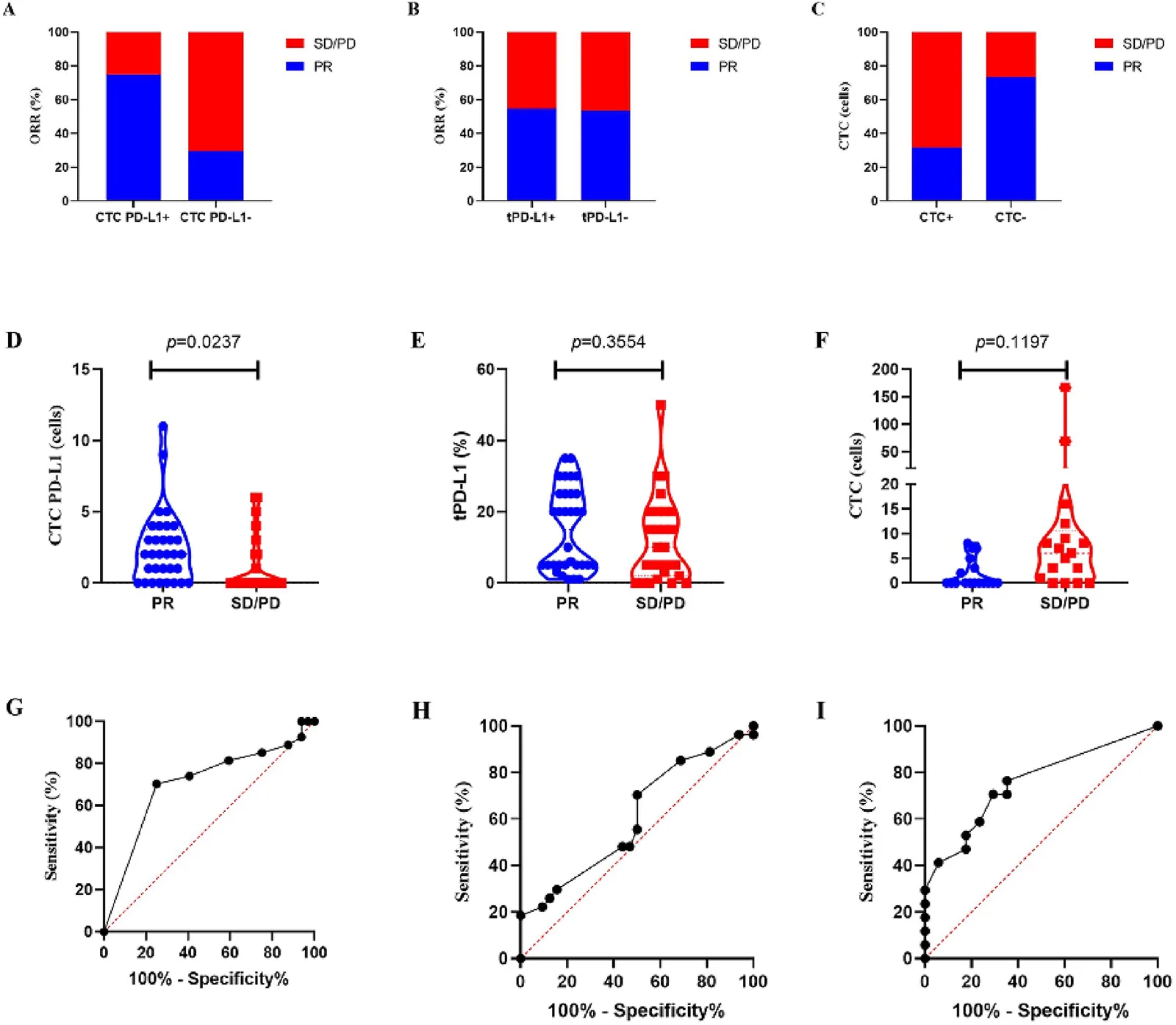
**Predictive value of CTC PD-L1, tumor tissue PD-L1, and total CTC counts for immunotherapeutic response.** (A–C) Stacked bar charts showing the ORR stratified by (A) CTC PD-L1 status (positive vs. negative), (B) tissue PD-L1 expression (positive vs. negative), and (C) CTC positivity (CTC + vs. CTC−). Blue and red bars represent PR and SD/PD, respectively. (D–F) Violin plots comparing the distributions of (D) CTC PD-L1 counts, (E) tissue PD-L1 expression percentage, and (F) total CTC counts between PR and SD/PD groups. Statistical comparisons were performed using the Mann–Whitney *U* test, with *p*-values of 0.0237, 0.3554, and 0.1197, respectively. (G–I) Receiver operating characteristic (ROC) curves evaluating the discriminative performance of (G) CTC PD-L1 counts, (H) tissue PD-L1 expression, and (I) total CTC count for differentiating responders (PR) from non-responders (SD/PD).

We next performed quantitative comparisons of the three candidate biomarkers between responders (PR) and non-responders (SD/PD) using violin plots. The absolute count of PD-L1-expressing CTCs was significantly higher in PR patients than in SD/PD patients (*p* = 0.0237; Fig. 3D). By contrast, no statistically significant intergroup difference was observed for tPD-L1 expression percentage (*p* = 0.3554; Fig. 3E). Although total CTC counts trended higher in the SD/PD cohort, this difference did not reach statistical significance (*p* = 0.1197; Fig. 3F).

To evaluate the predictive capacity of each biomarker for immunotherapeutic response, ROC curve analyses were performed (Fig. 3G–I). The ROC curve for CTC PD-L1 counts demonstrated moderate discriminative ability for distinguishing PR from SD/PD patients, with a sensitivity of 70.37% and specificity of 75.00% at a cutoff of 1 (AUC = 0.7049, 95% CI: 0.4605–0.7525, *p* = 0.0037; Fig. 3G). tPD-L1 expression failed to show significant predictive value, with an AUC of 0.6065 (95% CI: 0.5652–0.8445, *p* = 0.1616), a sensitivity of 70.37%, and a specificity of 50.00% at a cutoff of 12.5% (Fig. 3H). Total CTC count yielded an AUC of 0.7561 (95% CI: 0.5919–0.9202, *p* = 0.0108), with a sensitivity of 70.59% and specificity of 70.59% at a cutoff of 3 (Fig. 3I); however, its predictive efficiency was not superior to that of CTC PD-L1 counts when comprehensively evaluating the three ROC curves. Collectively, these data indicate that circulating PD-L1-positive CTC load, rather than tPD-L1 or total CTC enumeration, is significantly associated with favorable immunotherapy response and may serve as a more reliable predictive biomarker for therapeutic efficacy.

Moreover, we analyzed the association between each baseline clinicopathological factor and ORR (Table 2). No significant differences were observed between PR and SD/PD patients with respect to age (*p* = 0.277), gender (*p* = 0.197), cancer type (*p* = 0.227), clinical stage (*p* = 0.519), tumor size (*p* = 0.739), metastasis status (*p* = 0.506), or tissue PD-L1 expression level (*p* = 0.922), indicating that these conventional factors were well balanced between the two response groups. In marked contrast, CTC PD-L1 status exhibited a strong and statistically significant association with immunotherapeutic response. Among PR patients, 24 (75.0%) were CTC PD-L1-positive, whereas only 8 (29.6%) of SD/PD patients harbored CTC PD-L1-positive cells (*p* < 0.001), demonstrating a pronounced enrichment of baseline CTC PD-L1 positivity in responders relative to non-responders. Furthermore, although CTC positivity was also more frequently observed in the PR group (81.3%, 26/32) than in the SD/PD group (66.7%, 18/27), this difference did not reach statistical significance (*p* = 0.200).

**Table 2:** Baseline clinicopathological characteristics of patients stratified by immunotherapy response (PR vs. SD/PD) (n = 59).

	PR = 1	SD/PD = 0	*p*
Age	68.16 ± 10.896	71.00 ± 8.598	0.277
Gender			0.197
Female	8	11	
Male	24	16	
Cancer type			0.227
Adenocarcinoma	4	1	
Squamous cell carcinoma	28	26	
Stage			0.519
III	13	15	
IV	19	12	
Tumor size			0.739
T1-T2	12	9	
T3-T4	20	18	
Metastasis			0.506
M0	15	15	
M1	17	12	
Tissue PD-L1			0.922
<10%	15	13	
≥10%	17	14	
CTC PD-L1			<0.001*
Positive	24	8	
Negative	8	19	
CTC			0.200
Positive	26	18	
Negative	6	9	

* Significant relevance at *p* < 0.05.

Collectively, among all baseline variables examined, CTC PD-L1 status emerged as the only factor significantly associated with favorable response to chemo-immunotherapy in this cohort, underscoring its potential utility as a predictive biomarker for patient stratification prior to immunotherapy.

3. **Survival Analysis of Treatment Modalities, CTC Burden and PD-L1 Biomarkers**

Kaplan–Meier survival analyses were performed in the chemo-immunotherapy cohort (n = 59) to evaluate the prognostic value of baseline CTC counts, CTC PD-L1 expression, and tissue-derived tPD-L1 status. Patients receiving chemotherapy alone (n = 34) were analyzed separately as an exploratory reference group.

For PFS (Fig. 4A), CTC PD-L1-positive patients showed a non-significant trend toward better outcomes than CTC PD-L1-negative patients (median: 17.9 vs. 8.6 months; HR = 0.5247, 95% CI: 0.2672–1.030, *p* = 0.0610). For OS (Fig. 4B), CTC PD-L1-positive patients exhibited significantly superior survival compared with CTC PD-L1-negative patients (median: not reached vs. 20.4 months; HR = 0.2649, 95% CI: 0.1143–0.6141, *p* = 0.0010).

**Fig. 4:**
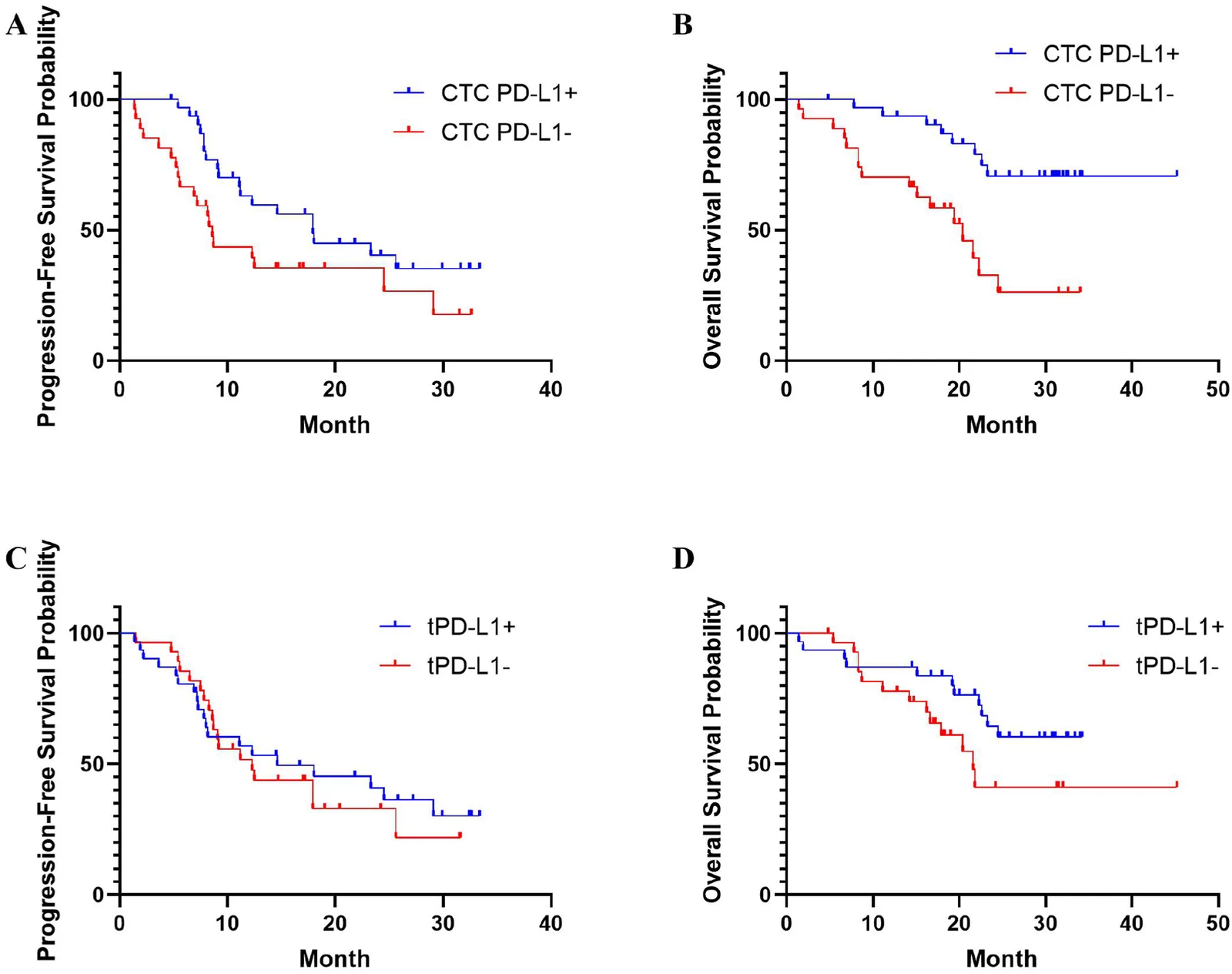
**Kaplan–Meier survival curves for PFS and OS stratified by treatment regimens and liquid/tissue PD-L1 biomarkers.** (A–D) Kaplan–Meier curves comparing patient outcomes across different subgroups, with PFS presented in the left column (A, C) and OS in the right column (B, D). (A, B) Curves grouped by CTC PD-L1 expression (CTC PD-L1+ in blue vs. CTC PD-L1− in red), with PFS (A) and OS (B) follow-up up to 50 months. (C, D) Curves stratified by tumor tissue PD-L1 expression (tPD-L1+ in blue vs. tPD-L1− in red), with PFS (C) and OS (D) follow-up up to 50 months.

For PFS (Fig. 4C), no significant difference was observed between tPD-L1-positive and tPD-L1-negative patients (Median: 14.6 vs. 12.3 months; HR = 0.8725, 95% CI: 0.4522–1.684, *p* = 0.6843). For OS (Fig. 4D), although tPD-L1-positive patients showed a numerically improved median survival (not reached vs. 21.6 months), this difference was not statistically significant (HR = 0.5319, 95% CI: 0.2339–1.210, *p* = 0.1103).

In an exploratory comparison, survival outcomes were also compared between patients receiving chemo-immunotherapy versus chemotherapy alone. For PFS (Supplementary Fig. S2A), the chemo-immunotherapy group demonstrated significantly prolonged survival compared with the chemotherapy-alone group (Median: 12.5 vs. 8.5 months; HR = 0.3866, 95% CI: 0.2236–0.6685, *p* = 0.0007). For OS (Supplementary Fig. S2B), the median survival in the chemo-immunotherapy group was not reached, versus 19.6 months in the chemotherapy-alone group, with a marginal but statistically significant difference (HR = 0.5463, 95% CI: 0.2761–1.081, *p* = 0.0474). Stratification by baseline CTC status was then performed to assess the prognostic impact of circulating tumor burden at chemotherapy group (n = 37). CTC-negative patients experienced significantly longer PFS (median: 13.2 vs. 6.7 months; HR = 0.2531, 95% CI: 0.1116–0.5742, *p* = 0.0010; Supplementary Fig. S2C) and longer OS (median: 28.8 vs. 19.40 months) (HR = 0.3006, 95% CI: 0.1022–0.8847, *p* = 0.0291; Supplementary Fig. S2D) than CTC-positive patients.

Univariate Cox proportional hazards regression analyses were performed to identify potential prognostic factors for PFS and OS (Table 3). Consistently with the Kaplan–Meier findings, CTC PD-L1 status was associated with PFS (HR = 0.546, *p* = 0.042) and OS (HR = 0.254, *p* = 0.002) in univariate analysis. In multivariable analysis (Table 4), CTC PD-L1 status also remained independently associated PFS (HR = 0.488, *p* = 0.039) and OS (HR = 0.244, *p* = 0.002). None of the other variables—including age, gender, clinical stage, metastasis status, tPD-L1 expression, or baseline CTC positivity—achieved statistical significance in either PFS or OS analyses (all *p* > 0.05).

**Table 3:** Univariate cox regression analysis of factors associated with progression-free survival and overall survival (n = 59).

Factors	PFS		OS	
	HR	*p* value	HR	*p* value
Age	1.019 (0.986-1.053)	0.259	1.018 (0.976-1.061)	0.408
Gender	0.921 (0.468-1.821)	0.811	0.804 (0.352-1.839)	0.606
Stage	1.164 (0.750-1.807)	0.499	0.839 (0.509-1.383)	0.490
Metastasis	1.141 (0.594-2.194)	0.692	1.361 (0.326-1.655)	0.457
tPD-L1	0.677 (0.453-1.672)	0.677	0.517 (0.228-1.174)	0.115
CTC	1.136 (0.535-2.411)	0.740	1.349 (0.604-3.013)	0.466
CTC PD-L1	0.546 (0.285-1.044)	0.042*	0.254 (0.107-0.602)	0.002*

* Significant relevance at *p* < 0.05.

**Table 4:** Multivariable cox regression analysis of factors associated with progression-free survival and overall survival (n = 59).

Factors	PFS		OS	
	HR	*p* value	HR	*p* value
Age	1.019 (0.983-1.057)	0.313	1.021 (0.977-1.066)	0.363
Gender	0.946 (0.468-1.910)	0.877	0.796 (0.337-1.883)	0.604
Stage	1.164 (0.750-1.807)	0.499	1.091 (0.502-2.371)	0.826
Metastasis	1.408 (0.246-2.044)	0.403	1.961 (0.556-6.900)	0.457
tPD-L1	0.962 (0.481-1.922)	0.912	0.540 (0.217-1.345)	0.540
CTC	1.450 (0.718-2.929)	0.300	1.350 (0.566-3.218)	0.498
CTC PD-L1	0.488 (0.247-0.966)	0.039*	0.244 (0.100-0.595)	0.002*

* Significant relevance at *p* < 0.05.

Collectively, both Kaplan–Meier and multivariable Cox regression analyses consistently identified chemo-immunotherapy and CTC PD-L1 status as the two most robust prognostic factors for OS, whereas conventional clinicopathological parameters and tissue-based tPD-L1 expression showed limited predictive value for patient survival.

## 4 Discussion

In this study, we comprehensively evaluated the clinical utility of CTC PD-L1 expression as a predictive and prognostic biomarker in patients with stage III–IV esophageal cancer receiving immunotherapy-based treatment. Our findings demonstrated that baseline CTC PD-L1 status was significantly associated with both ORR and OS, and appeared to perform better than conventional tissue-based tPD-L1 testing and total CTC enumeration in this cohort.

The prognostic value of CTCs in various malignancies has been well documented, with detectable CTCs generally indicating aggressive tumor behavior and poor clinical outcomes [22], [23], [24]. In our cohort, baseline CTC positivity was associated with advanced T stage and metastatic status, consistent with the established role of CTCs as surrogates of tumor dissemination. However, total CTC count alone showed limited predictive power for immunotherapeutic response and survival in multivariate analyses, underscoring that mere presence of CTCs is insufficient to guide immunotherapy decisions. This observation aligns with previous reports suggesting that the phenotypic characterization of CTCs, rather than their enumeration, provides more clinically relevant information [25], [26].

Among the biomarkers examined, CTC PD-L1 expression emerged as the most robust predictor of favorable clinical outcomes. Patients with CTC PD-L1-positive status achieved significantly higher ORR (75.0% vs. 29.6%) and markedly prolonged PFS and OS compared with CTC PD-L1-negative individuals. Notably, this association remained highly significant in both Kaplan–Meier survival analyses and univariate Cox regression, indicating a substantially reduced risk of disease progression and death. The strong prognostic performance of CTC PD-L1 may be attributed to its ability to capture the real-time PD-L1 status of circulating tumor cells, which reflects the dynamic interplay between tumor cells and the host immune system [27], [28], [29].

In marked contrast, tissue-based tPD-L1 expression, as determined by standard immunohistochemistry, failed to demonstrate significant associations with ORR, PFS, or OS in our study. Although tPD-L1 positivity showed a numerical trend toward improved survival, neither the Kaplan–Meier nor the Cox regression analyses reached statistical significance. The discrepancy between tissue and circulating PD-L1 assessments is clinically intriguing. Several factors may account for this observation. First, tumor tissue PD-L1 expression is subject to spatial heterogeneity, and a single biopsy specimen may not fully represent the overall PD-L1 status of the tumor [10], [12]. Second, PD-L1 expression is dynamic and can change in response to therapeutic interventions and evolving immune pressure [30], [31]. CTCs are shed from both primary and metastatic sites and may therefore provide a more integrated assessment of the tumor's PD-L1 landscape [32], [33]; however, whether CTC PD-L1 truly overcomes intratumoral heterogeneity remains to be directly demonstrated, and our data do not establish this conclusively. Furthermore, CTCs can be serially monitored through repeated blood draws, enabling real-time tracking of PD-L1 dynamics and early detection of resistance mechanisms.

The superior predictive performance of CTC PD-L1 over total CTC count is another important finding. While total CTC count trended higher in non-responders and in patients with poor survival outcomes, these differences did not reach statistical significance in most analyses. In contrast, CTC PD-L1 status consistently stratified patients into distinct prognostic groups for PFS and OS. This observation suggests that the functional state of CTCs may be more biologically relevant than their absolute number. PD-L1-positive CTCs are thought to mediate immune evasion through direct engagement with PD-1-expressing T cells in the circulation, thereby suppressing antitumor immunity and promoting tumor progression [34]. Therefore, the presence of PD-L1-expressing CTCs may reflect an active immune-escape phenotype that directly impacts therapeutic outcomes.

The prognostic value of treatment modality was also confirmed in our study, with chemo-immunotherapy demonstrating significantly improved PFS and OS compared with chemotherapy alone. This finding is consistent with the growing body of evidence supporting the addition of immune checkpoint inhibitors to standard chemotherapy as first-line treatment for esophageal cancer [35], [36]. Importantly, the survival benefit of chemo-immunotherapy was observed across both short-term and extended follow-up windows, indicating a durable clinical advantage. However, not all patients derived equal benefit from this combination strategy, underscoring the critical need for reliable predictive biomarkers to guide treatment selection. Our results suggest that CTC PD-L1 status could help identify patients most likely to respond to chemo-immunotherapy, thereby enabling more personalized treatment decisions.

Several additional limitations should be acknowledged. First, the sample size was relatively modest (N = 93; chemo-immunotherapy cohort n = 59), limiting statistical power, particularly for PFS and subgroup analyses. Second, the Mx category included patients with unknown or unassessed metastatic status, which may have introduced heterogeneity. Third, the CTC PD-L1 detection method requires further standardization, and data on analytical variability and inter-observer agreement, although assessed here, need confirmation in larger multi-center settings. Fourth, the observational nature of the study precludes causal inferences, and treatment allocation was non-random, so selection bias cannot be excluded. Fifth, although multivariable Cox regression was performed, residual confounding may remain, and the number of events limited the number of variables that could be included. Finally, direct evidence that CTC PD-L1 overcomes tissue heterogeneity is lacking, and our findings should be regarded as exploratory.

In conclusion, our findings demonstrate that baseline CTC PD-L1 expression is associated with favorable immunotherapeutic response and improved survival outcomes in patients with esophageal cancer. Compared with tissue-based tPD-L1 testing and total CTC enumeration, CTC PD-L1 assessment may provide better prognostic stratification and may serve as a potentially useful biomarker for guiding immunotherapy decisions. These exploratory findings require prospective validation in larger, multi-center cohorts.

## Ethics declaration

Written informed consent to take part in the study and to publish the article has been obtained from all participants or their legal representatives. The privacy rights of participants have been observed.

This study included organ or tissue donors.This study includes human biological material and consent was obtained by donors, or their next of kin or legal representatives, for use in this study and for publication of the article. The samples used in this research were not sourced from executed prisoners or prisoners of conscience.

This study was performed in compliance with relevant laws, regulatory frameworks and guidelines where the research took place.This study was conducted in accordance with 1964 Helsinki Declaration.This study was approved by the First Affiliated Hospital of Zhengzhou University.(Approval No. 2024-KY-0100-002)

## Ethics approval statement

This study is approved by the Medical Ethics Committee of the First Affiliated Hospital of Zhengzhou University, with ethics approval number 2024-KY-0100-002 on April, 2024. All procedures involving human participants and human tissue samples were conducted in accordance with the 1964 Helsinki Declaration and its later amendments or comparable ethical standards.

## Consent for publication

Written informed consent for publication was obtained from the patients or legal guardian.

## Availability of data and materials

All data and materials relevant to the study are included in the article.

## Authors contributions

**Jing Li, Yanru Qin, and Dongjiang Tang:** concepted, designed and supervised the study; drafted and critically revised the manuscript. **Yueze Li:** responsible for ethical applications, patient screening and enrollment, clinical outcomes of samples, and statistical analysis. **Lin Chen:** designed and performed most of the experiments; acquired, analyzed and interpreted data; and drafted the manuscript. **Zhonglin Yang and Yue Lu:** provided technical support.

## Funding

The study was supported by 10.13039/501100003009Macao Science and Technology Development Fund (FDCT: 0009/2022/AKP).

## Declaration of competing interest

The authors declare that they have no known competing financial interests or personal relationships that could have appeared to influence the work reported in this paper.
