# Supplementary material for: Clinical utility of circulating tumor cell PD-L1 expression for predicting immunotherapeutic response and prognosis in esophageal cancer
Source: J Liq Biopsy. 2026 Oct 21;14:100681. doi: 10.1016/j.jlb.2026.100681 (PMC13715408; doi:10.1016/j.jlb.2026.100681)
Supplement: Supplementary file 1 [file mmc1.doc]

**Supplementary Table S1: Median, range and IQR of CTC and CTC PD-L1 in overall cohort and subgroups.**

| Subgroups | CTC | Median | Q1 | Q2 | IQR | Range |
| --- | --- | --- | --- | --- | --- | --- |
| Overall cohort | CTC | 3 | 1 | 5 | 4 | 0-167 |
|  | CTC PD-L1 | 1 | 0 | 3 | 3 | 0-159 |
| Male | CTC | 3 | 0 | 6 | 6 | 0-16 |
|  | CTC PD-L1 | 1 | 0 | 3 | 3 | 0-11 |
| Female | CTC | 3 | 0 | 6 | 6 | 0-167 |
|  | CTC PD-L1 | 0.5 | 0 | 2 | 2 | 0-159 |
| Adenocarcinoma | CTC | 3 | 0 | 5 | 5 | 0-5 |
|  | CTC PD-L1 | 0 | 0 | 3 | 3 | 0-5 |
| Squamous cell carcinoma | CTC | 3 | 1 | 6 | 5 | 0-167 |
|  | CTC PD-L1 | 1 | 0 | 3 | 3 | 0-159 |
| Stage III | CTC | 1 | 0 | 3 | 3 | 0-7 |
|  | CTC PD-L1 | 0 | 0 | 1 | 1 | 0-6 |
| Stage IV | CTC | 4 | 1 | 7 | 6 | 0-167 |
|  | CTC PD-L1 | 2 | 0 | 4 | 4 | 0-159 |
| Metastasis | CTC | 4 | 1 | 7 | 6 | 0-167 |
|  | CTC PD-L1 | 2 | 0 | 4 | 4 | 0-159 |
| Non metastasis | CTC | 1 | 0 | 3 | 3 | 0-7 |
|  | CTC PD-L1 | 0 | 0 | 1 | 1 | 0-6 |
| PR | CTC | 2 | 0 | 5 | 5 | 0-11 |
|  | CTC PD-L1 | 1 | 0 | 3 | 3 | 0-11 |
| SD/PD | CTC | 3 | 0 | 6 | 6 | 0-167 |
|  | CTC PD-L1 | 0 | 0 | 3 | 3 | 0-159 |

**Supplementary Table S2: Correlation analysis of tPD-L1 to CTC/CTC PD-L1.**

| **Variable 1** | **Variable 2** | **Spearman's rho** | **p-value** |
| --- | --- | --- | --- |
| tPD-L1 | CTC | 0.091 | 0.396 |
| tPD-L1 | CTC PD-L1 | 0.136 | 0.200 |

* Significant relevance at *p*<0.05

**Supplementary Figure S2. Kaplan–Meier analysis of PFS and OS stratified by chemo-immunotherapy versus chemotherapy alone and by baseline CTC status.** (A, B) Curves comparing chemo‑immunotherapy (blue) versus chemotherapy alone (red), with PFS (A) and OS (B) follow‑up up to 40 months. (C, D) Curves stratified by baseline CTC status (CTC+ in blue vs. CTC− in red), with PFS (C) within 25 months and OS (D) extended to 40 months.
